# Effectiveness of an e-Book on Bone Health as Educational Material for Adolescents: Single-Group Experimental Study

**DOI:** 10.2196/56611

**Published:** 2024-08-26

**Authors:** Nor Aini Jamil, Jashwiny Dhanaseelan, Nurin Athirah Buhari

**Affiliations:** 1 Centre for Community Health Studies, Faculty of Health Sciences, Universiti Kebangsaan Malaysia, Jalan Raja Muda Abd Aziz, Kuala Lumpur, 50300, Malaysia, 60 392897081

**Keywords:** osteoporosis, bone health, adolescent, knowledge, calcium, physical activity, e-book, effectiveness, educational, teens, youth, bone fragility, bone, Malaysia, online questionnaire, sociodemographic, calcium intake, diet, behavior change

## Abstract

**Background:**

Improved bone health during adolescence can have lifelong implications, reducing the risk of bone fragility.

**Objective:**

This study aims to evaluate the effectiveness of an e-book in increasing knowledge about and promoting healthy practices related to bone health among Malay adolescents in Kuala Lumpur, Malaysia.

**Methods:**

A total of 72 adolescents (female: n=51, 71%; age: mean 15, SD 0.74 y) were recruited from selected secondary schools. The participants answered a pretest web-based questionnaire on sociodemographic data, knowledge about osteoporosis, and physical activity. A video call was conducted to assess dietary calcium intake. Participants were provided with a link to an e-book on bone health and instructed to read it within 2 weeks. Postintervention assessments included those for knowledge, physical activity, dietary calcium intake, and acceptance of the e-book.

**Results:**

There was a significant increase in the median knowledge score, which was 40.6% (IQR 31.3%-46.9%) during the pretest and 71.9% (IQR 53.9%-81.3%) during the posttest (*P*<.001). However, no changes were observed in dietary calcium intake or physical activity levels. Most participants did not meet the recommended calcium requirements (61/62, 98%) and exhibited sedentary behavior (pretest: 51/62, 82%; posttest: 48/62, 77%). The e-book, however, was well accepted, with the majority reporting that they understood the contents (70/72, 97%), liked the graphics (71/72, 99%), and approved of the layout (60/72, 83%) and font size (66/72, 92%) used.

**Conclusions:**

The developed e-book effectively increases knowledge levels related to bone health and is well accepted among participants. However, this educational material did not improve bone health practices. Additional strategies are necessary to bridge the gap between knowledge and behavior change.

## Introduction

Throughout the human life span, bone modelling and remodelling are continuous and vital processes that shape bone architecture, crucially contributing to peak bone mass attainment [1]. Childhood and adolescence mark crucial periods for rapid bone growth and peak bone mass—the highest bone mineral density (BMD) attained in one’s lifetime [2]. Improving bone health during these phases yields long-term benefits, reducing the risk of bone fragility and fractures later in life [3]. Since BMD typically declines with age, achieving peak bone mass by 30 years of age and maintaining bone density into middle age are pivotal for mitigating osteoporosis risk [4]. Notably, a 10% increase in peak bone mass can delay the onset of osteoporosis by up to 13 years [5]. Factors that influence peak bone mass include genetics, calcium and vitamin D intake, and physical activity [6].

Osteoporosis is a systemic bone disease characterized by reduced bone mass and microstructural deterioration, heightening bone fragility and fracture susceptibility [7]. The disease results from an imbalance between bone formation and resorption, with the latter predominating. Osteoporosis prevalence generally increases with age [8], affecting approximately 1 in 3 women and 1 in 5 men aged older than 50 years [7]. A systematic review and meta-analysis estimated the global prevalence of osteoporosis to be 18.3%, based on a cohort of 103,334,579 individuals [9]. The analysis encompassed 86 studies that involved participants aged 15 to 105 years [9]. In Kuala Lumpur, Malaysia, a cross-sectional study reported a prevalence of 12.3% among 786 adults aged 40 years and older [10]. By 2050, the hip fracture incidence in Malaysia is projected to increase to 3.55 times that in 2018 [11]. In China, a study involving 9826 women aged 40 years and older reported a slightly higher osteoporosis prevalence of 20.6% [12], while Korea showed a prevalence of 38% among women aged 50 years and older in a sample of 4011 individuals [13]. Preventive measures against osteoporosis include optimizing peak bone mass during adolescence, maintaining bone density in adulthood, and mitigating age-related bone loss [14,15].

Lifestyle factors, such as dietary calcium intake, vitamin D status, and weight-bearing exercise, significantly influence bone health. Calcium plays a crucial role in maximizing bone density and strength during growth and development [6]. Adequate calcium intake is important for achieving optimal peak bone mass and promoting bone health, whereas insufficient intake increases the risk of low BMD [16]. Vitamin D, which is synthesized through skin exposure to UV-B rays and obtained from dietary sources, is essential for calcium homeostasis and absorption [17,18]. Insufficient vitamin D status is a global concern and is exacerbated by environmental factors, clothing practices, sunscreen use, and weather variations [19]. High-impact and weight-bearing exercises, such as running and jumping, enhance bone strength [20]. However, studies in Malaysia have identified suboptimal dietary calcium intakes [21,22], insufficient vitamin D status [21-25], and low physical activity levels among adolescents [26].

Raising osteoporosis awareness among adolescents is important for reducing the risk of the disease later in life [27]. Despite its significance, research on osteoporosis knowledge among adolescents in Malaysia is limited, with such research predominantly focusing on adults and older populations [10,28]. One study highlighted deficiencies in osteoporosis knowledge and engagement in bone health practices among adolescents [29]. Specifically, adolescents and young adults often perceive other diseases as more serious than osteoporosis [29]. This underscores the need to increase awareness of bone health and osteoporosis among adolescents.

Educational programs targeting osteoporosis prevention hold significant potential for improving adolescent bone health [30]. A systematic review and meta-analysis conducted by Abdolalipour and Mirghafourvand [30] demonstrated that such programs positively influence adolescent osteoporosis prevention by improving dietary calcium intake, promoting preventive practices, and enhancing knowledge about and attitudes toward osteoporosis. Therefore, implementing initiatives that focus on adolescent osteoporosis prevention is paramount for fostering healthy lifestyles. Research consistently demonstrates that these educational interventions enhance knowledge, shape attitudes, and encourage dietary practices conducive to bone health [30]. In-depth individual and semistructured interviews with 60 young adults aged 17 to 30 years highlighted that while most seek bone health information on the internet, traditional sources such as parents, doctors, educators, and peers remain valuable [31]. Importantly, participants preferred educational programs that deliver concise and relatable messages and promote gradual lifestyle changes [31].

e-Books use computer technology to deliver information dynamically through sounds, graphics, images, animations, and videos, offering enriched content when compared to conventional books [32]. A literature review that assessed the effectiveness of e-books in student learning reported high effectiveness scores, which ranged from 80% to over 95% across multiple studies [32]. Furthermore, a study that compared e-books to traditional PowerPoint (Microsoft Corporation) methods in clinical teaching found that e-books enhance student satisfaction, improve teaching quality and methods, and increase student engagement [33]. This study aims to assess the effectiveness of an e-book with interactive multimedia features in increasing knowledge about and promoting healthy practices related to bone health among Malay adolescents in Kuala Lumpur, Malaysia.

## Methods

### Study Design and Participants

This study used an experimental design with purposive sampling to gather data from Malay adolescents aged 14 to 16 years who attended selected secondary schools in Kuala Lumpur, Malaysia. Without a control group, the chosen single-group experimental approach ensured uniform access to the educational material among all participants, aligning with ethical and practical considerations in academic research. Practical constraints within the school settings, such as challenges in recruiting a comparable control group and ensuring their nonexposure to the e-book, further supported the selection of this design. Despite inherent limitations in terms of internal validity, particularly threats related to the absence of a control group, this study represents an initial exploration of e-books as educational tools for adolescents.

Initially, invitations were extended to 14 schools, with 6 granting permission for participation following the dissemination of relevant information to homeroom teachers. Eligible participants were required to have access to electronic devices, such as mobile phones, tablets, or computers, with internet connectivity. Adolescents with health conditions, including visual or hearing impairments, autism spectrum disorder, attention-deficit/hyperactivity disorder, Down syndrome, cerebral palsy, dyslexia, aphasia, or physical disabilities, were excluded from this study.

### Ethical Considerations

Ethical approval was obtained from the Universiti Kebangsaan Malaysia’s research ethics committee (reference code: UKM PPI/111/8/JEP-2022‐139). The data collected in this study were anonymized by using a study-specific identification number. Further, participants were given a notebook worth RM 5 (US $1.12) as a token of appreciation.

### Data Collection

The research information sheet and an informed consent and screening form were disseminated through a web-based platform (WhatsApp [Meta Platforms] messages with a document link) by homeroom teachers. Interested participants who met the eligibility criteria and obtained parental or guardian consent were contacted and provided with a link to access the pretest questionnaire via Google Forms. This questionnaire gathered sociodemographic information, assessed knowledge about osteoporosis, and collected data on physical activity levels.

After completing the pretest questionnaire, a video call session via Google Meet was scheduled to assess habitual dietary calcium intake. Participants were subsequently given access to an e-book on bone health and instructed to read it within 2 weeks. At the end of this period, participants completed a posttest questionnaire, which was almost identical to the pretest questionnaire but excluded sociodemographic information. Additionally, the posttest questionnaire included a section to assess participants’ acceptance of the e-book. A second video call session via Google Meet was conducted to reassess participants’ dietary calcium intake. The entire data collection process was conducted via the internet to minimize interference with school schedules.

### Research Instruments

#### e-Book

The e-book, which consisted of 58 pages, was previously developed and validated by an expert panel [34]. It featured vibrant infographics and illustrations that covered the following seven subtopics: (1) “Let’s Know Our Bones,” (2) “Osteoporosis,” (3) “Adolescents’ Bone Health,” (4) “Calcium,” (5) “Vitamin D,” (6) “Physical Activity & Exercise for Bone Strength,” and (7) “7 Healthy Lifestyles for Bone Health.” In addition, the e-book included a 5-minute summary video and a link to a short quiz.

#### Sociodemographic Profiles

The sociodemographic data collected included sex, date of birth, household income, and prior participation in a bone health or osteoporosis education program. Participants self-reported their body weight and height, and the BMI was calculated by dividing weight by the square of height. BMI-for-age was analyzed by using WHO (World Health Organization) AnthroPlus version 1.0.4 (WHO), with classifications based on WHO cutoff points [35].

#### Osteoporosis Knowledge

Participants’ knowledge about bone health and osteoporosis was assessed by using the Revised Osteoporosis Knowledge Test Questionnaire, which has demonstrated high internal consistency (Kuder-Richardson-20 values of 0.85 overall, 0.83 for the nutrition subscale, and 0.81 for the exercise subscale) and test-retest reliability (*r*=0.87) [36]. The questionnaire was confirmed through content validity, establishing it as a comprehensive instrument that reflects current research and assesses osteoporosis knowledge in adults [36]. We adapted this questionnaire to assess osteoporosis knowledge in adolescents. A previous study used this questionnaire to determine osteoporosis knowledge in Iranian adolescents [37].

The 32-item questionnaire was translated into Malay by using a back-translation method. Some questions (eg, those concerning the recommended amount of calcium and vitamin D intake) were modified to align with Malaysian dietary guidelines for adolescents [16]. One point was given for each correct answer, and 0 points were given for incorrect or “Do not know” responses. Each participant’s total score was then converted into a percentage and classified as a high (>80%), medium (60%‐80%), or low (<60%) knowledge level based on previous osteoporosis research [38].

#### Practices Related to Bone Health

Dietary calcium intake was assessed by using a food frequency questionnaire (FFQ) adapted from a previous study on postmenopausal women in Malaysia [39]. Notably, there was limited availability of FFQs tailored for adolescents in Malaysia. This FFQ, which moderately correlated with a 3-day food record (*r*=0.563) [39], encompassed 78 food items. Administration of the FFQ was conducted through web-based video call interviews. Participants reported their frequency of consumption (daily, weekly, or monthly) and estimated portion sizes for each food item. The total calcium intake for each participant was calculated by multiplying frequency of consumption; portion sizes (g); and calcium content per 100 g, which was derived from the Malaysian food database [40].

Physical activity levels were assessed by using the Physical Activity Questionnaire for Adolescents (PAQ-A) [41]. The questionnaire was previously translated into Malay and demonstrated a low correlation (*r*=0.23; *P*=.08) but high reliability (*r*=0.72; *P*<.001) when compared to pedometer data [42]. A study conducted in Poland demonstrated a strong correlation between PAQ-A scores and accelerometer data (*r*=0.94; *P*<.01) [43].

The PAQ-A consists of 9 items that evaluate the participants’ physical activity involvement over the past 7 days. Each item is rated on a 5-point scale, ranging from 1 (indicating a low level of physical activity) to 5 (indicating a high level of physical activity).

#### Acceptance of the e-Book

Acceptance of the e-book was evaluated by using a questionnaire adapted from a study that assessed physical information leaflets for pregnant women [44]. The assessment focused on the following three domains: content, graphics, and design. The level of acceptance was determined based on the percentage of responses within each domain. Although the instrument was initially developed for evaluating physical information leaflets (ie, it may not directly align with e-books), the assessed domains (content, graphics, and design) remain pertinent for evaluating educational materials. In this study, acceptance of the e-book served as a secondary outcome.

### Statistical Analysis

Data were analyzed by using IBM SPSS Statistics software (version 26.0; IBM Corp). The normality of continuous data was assessed by using the Shapiro-Wilk test. Descriptive statistics were reported as means and SDs for normally distributed data or medians and IQRs for nonnormally distributed data. Categorical variables were summarized as frequencies and percentages. The Wilcoxon signed rank test was used to compare pre- and postintervention scores of knowledge, physical activity, and calcium intake, due to the nonnormal distribution of the data. For categorical variables, associations and comparisons were assessed by using the chi-square test or McNemar test. A *P* value of <.05 was considered statistically significant.

## Results

### Sociodemographic Profiles

A total of 72 participants (age: mean 15, SD 0.74 y) were enrolled in this study, and no dropouts were recorded (Table 1). The majority of the participants were female (n=51, 71%). The household income distribution was nearly evenly split among the low-income (n=23, 32%), middle-income (n=23, 32%), and high-income (n=26, 36%) categories. Most participants (n=51, 71%) were within the normal weight range, with a mean BMI of 20.9 (SD 4.5) kg/m^2^. Only 1 participant reported prior education on bone health.

**Table 1.** Sociodemographic profiles and physical characteristics of participants (N=72).

Parameter		Value	
Age (y), mean (SD)		15.0 (0.74)	
**Sex, n (%)**			
	Male	21 (29)	
	Female	51 (71)	
**Household income (RM^a^), n (%)**			
	Low (<4850)	23 (32)	
	Middle (4850‐10,959)	23 (32)	
	High (>10,959)	26 (36)	
Weight (kg), mean (SD)		53.2 (13.3)	
Height (m), mean (SD)		1.59 (0.07)	
BMI (kg/m^2^), mean (SD)		20.9 (4.5)	
**BMI category, n (%)**			
	Thinness (<−2 SDs)	6 (8)	
	Normal (≥−2 SDs)	51 (71)	
	Overweight (>+1 SD)	9 (13)	
	Obese (>+2 SDs)	6 (8)	
Attending bone health education program, n (%)		1 (1)	

a A currency exchange rate of RM 1=US $0.22 is applicable.

### Osteoporosis Knowledge

The educational intervention significantly improved participants’ knowledge of osteoporosis; the median knowledge score increased from 40.6% (IQR 31.3%-46.9%) in the pretest to 71.9% (IQR 53.9%-81.3%) in the posttest (*P*<.001; Table 2). Before the intervention, the majority of the participants demonstrated low levels of knowledge (69/72, 96%). There was a notable improvement after the intervention, with 38% (27/72) achieving moderate knowledge and 32% (23/72) reaching a high level of knowledge.

Table 3 presents the percentages of correct and incorrect responses (“False” or “Do not know”) before and after exposure to the educational materials. Significant improvements were observed across all knowledge domains. Notably, 36% (26/72) of participants remained unaware that aging increases the risk of osteoporosis. Similarly, a small proportion of participants did not recognize the benefits of aerobic dancing for bone health (8/72, 11%), the fact that cheese is a good source of calcium (8/72, 11%), and that adolescence is a critical period for building strong bones (2/72, 3%).

**Table 2.** Osteoporosis knowledge before and after the educational intervention (N=72).

Osteoporosis knowledge		Pretest	Posttest	*P* value
Score (%), median (IQR)		40.6 (31.3-46.9)	71.9 (53.9-81.3)	<.001^a^
**Knowledge classification, n (%)**				.34^b^
	High (score: >80%)	0 (0)	23 (32)	
	Moderate (score: 60%‐80%)	3 (4)	27 (38)	
	Low (score: <60%)	69 (96)	22 (31)	

a Wilcoxon signed rank test.

b Chi-square test.

**Table 3.** Analysis of osteoporosis knowledge questions before and after the educational intervention (N=72).

Questions		Pretest (correct), n (%)	Posttest (correct), n (%)	Pretest (incorrect), n (%)	Posttest (incorrect), n (%)	*P* value
**Domain: risk factors of osteoporosis**						
	1. Eating a diet low in dairy products	40 (56)	54 (75)	32 (44)	18 (25)	.01
	2. Being menopausal; “change of life”	26 (36)	50 (69)	46 (64)	22 (31)	<.001^a^
	3. Having a parent or grandparent who has osteoporosis	29 (40)	56 (78)	43 (60)	16 (22)	<.001^a^
	4. Being a White or Asian woman	5 (7)	28 (39)	67 (93)	44 (61)	<.001^a^
	5. Being an elderly	43 (60)	46 (64)	29 (40)	26 (36)	.73
	6. Having ovaries surgically removed	10 (14)	41 (57)	62 (86)	31 (43)	<.001^a^
	7. Taking cortisone (steroids; eg, prednisone) for a long time	25 (35)	43 (60)	47 (65)	29 (40)	.001
	8. Being overweight	33 (46)	53 (74)	39 (54)	19 (26)	.001
	9. Having an eating disorder	34 (47)	53 (74)	38 (53)	19 (26)	.003
	10. Consuming more than 2 alcoholic drinks per day	37 (51)	62 (86)	35 (49)	10 (14)	<.001^a^
	11. Smoking on a daily basis	38 (53)	55 (76)	34 (47)	17 (24)	.003
**Domain: exercise**						
	12. To strengthen bones, it is recommended that a person exercises at a moderately intense level for 30 min a day at least.... (answer: 7 days a week)	3 (4)	26 (36)	69 (96)	46 (64)	<.001^a^
	13. Exercise makes bones strong, but it must be hard enough to make breathing.... (answer: much faster, but talking is possible)	35 (49)	54 (75)	37 (51)	18 (25)	.001
	14. Which of the following activities is the best way to reduce a person’s chance of getting osteoporosis? (answer: walking briskly)	6 (8)	25 (35)	66 (92)	47 (65)	<.001^a^
	15. Which of the following activities is the best way to reduce a person’s chance of getting osteoporosis? (answer: lifting weights)	8 (11)	28 (39)	64 (89)	44 (61)	<.001^a^
	16. Which of the following activities is the best way to reduce a person’s chance of getting osteoporosis? (answer: jogging or running)	58 (81)	70 (97)	14 (19)	2 (3)	.004
	17. Which of the following activities is the best way to reduce a person’s chance of getting osteoporosis? (answer: aerobic dancing)	59 (82)	64 (89)	13 (18)	8 (11)	.38
**Domain: calcium & vitamin D**						
	18. Which of these is the best source of calcium? (answer: cheese)	52 (72)	64 (89)	20 (28)	8 (11)	.12
	19. Which of these is the best source of calcium? (answer: canned sardines)	14 (19)	44 (61)	58 (81)	28 (39)	<.001^a^
	20. Which of these is the best source of calcium? (answer: broccoli)	37 (51)	53 (74)	35 (49)	19 (26)	.005
	21. Which of these is the best source of calcium? (answer: yogurt)	56 (78)	67 (93)	16 (22)	5 (7)	.003
	22. Which of these is the best source of calcium? (answer: ice cream)	6 (8)	35 (49)	66 (92)	37 (51)	<.001^a^
	23. Which of these is the best source of calcium? (answer: almonds)	19 (26)	39 (54)	53 (74)	33 (46)	.001
	24. Which of the following is the recommended amount of calcium intake for an adolescent? (answer: 1300 mg daily)	16 (22)	54 (75)	56 (78)	18 (25)	<.001^a^
	25. How much milk must an adolescent drink to meet the recommended amount of calcium? (answer: 3 or more glasses daily)	18 (25)	47 (65)	54 (75)	25 (35)	<.001^a^
	26. Which vitamin is required for absorption of calcium? (answer: vitamin D)	37 (51)	55 (76)	35 (49)	17 (24)	.003
	27. Which is the best source of the vitamin required for the absorption of calcium? (answer: sunlight)	25 (35)	51 (71)	47 (65)	21 (29)	<.001^a^
	28. Which is the best food source of the vitamin required for the absorption of calcium? (answer: salmon)	15 (21)	42 (58)	57 (79)	30 (42)	<.001^a^
	29. Which of the following is the recommended amount of the vitamin required for the absorption of calcium for an adolescent? (answer: 15 µg daily)	18 (25)	47 (65)	54 (75)	25 (35)	<.001^a^
**Domain: general knowledge on bone health**						
	30. When is the best time to build strong bones? (answer: adolescence)	67 (93)	70 (97)	5 (7)	2 (3)	.45
	31. Osteoporosis can be diagnosed by.... (answer: DXA^b^ scan)	42 (58)	58 (81)	30 (42)	14 (19)	.002
	32. Once you have osteoporosis.... (answer: you can take medication to treat it)	19 (26)	42 (58)	53 (74)	30 (42)	<.001^a^

a McNemar test.

b DXA: dual-energy x-ray absorptiometry.

### Practices Related to Bone Health

No significant differences were observed in dietary calcium intake (*P*=.31) and physical activity levels (*P*=.09) among adolescents following the intervention. Most participants exhibited inadequate calcium intake before (61/62, 98%) and after (61/62, 98%) the intervention. Similarly, a high proportion of participants maintained low levels of physical activity before (51/62, 82%) and after (48/62, 77%) the education intervention (Table 4).

**Table 4.** Practices related to bone health among participants (n=62).

Practices		Pretest	Posttest	*P* value
Calcium intake (mg), median (IQR)		575.8 (472.5-688.0)	585.9 (490.9-754.7)	.31^a^
Physical activity level (score), median (IQR)		2.2 (1.9-2.6)	2.3 (1.9-2.9)	.09^a^
**Calcium intake classification, n (%)**				>.99^b^
	Adequate (>1300 mg)	1 (2)	1 (2)	
	Inadequate (<1300 mg)	61 (98)	61 (98)	
**Physical activity level classification, n (%)**				.53^b^
	High (score: 4‐5)	2 (3)	2 (3)	
	Moderate (score: 3)	9 (15)	12 (19)	
	Low (score: 1‐2)	51 (82)	48 (77)	

a Wilcoxon signed rank test.

b McNemar test.

### Acceptance of the e-Book

Table 5 shows participants’ feedback on the e-book. Overall, 97% (70/72) reported understanding the e-book content, with 71% (63/89) of responses (for the “Reasons for good understanding” question, participants could choose multiple responses) indicating that simple and easy-to-understand language facilitated comprehension. Furthermore, 93% (67/72) of participants emphasized the importance of graphics in the e-book, and nearly all participants (71/72, 99%) found the visuals attractive. Additionally, 78% (56/72) agreed that the color combination enhanced their reading experience, while most adolescents considered the layout of the pictures (60/72, 83%) and the font size (66/72, 92%) to be appropriate.

**Table 5.** Acceptance of the e-book on bone health among participants (N=72).

Evaluation aspects		Participants, n (%)	
**Understanding**			
	Good understanding	70 (97)	
**Reasons for good understanding^a^**			
	Simple and easy-to-understand sentence	63 (71)	
	Appropriate words/terms used	17 (19)	
	Attractive e-book	4 (5)	
	Concise and precise	3 (3)	
	Many photos/diagrams	2 (2)	
**Graphics importance**			
	Yes	67 (93)	
**Attractiveness of graphic**			
	Attractive	71 (99)	
**Color influenced reading**			
	Yes	56 (78)	
**Appropriate picture layout**			
	Yes	60 (83)	
**Appropriate font size**			
	Yes	66 (92)	

a Participants could choose multiple responses for this particular question. As such, the n values here refer to the number of responses, and the percentages were calculated by using the total number of these responses as the denominator (N=89).

## Discussion

### Osteoporosis Knowledge

This study demonstrates the effectiveness of an e-book–based educational intervention in enhancing adolescents’ knowledge of bone health and osteoporosis. Our findings align with a meta-analysis of 18 studies, which reported significant differences in osteoporosis knowledge among adolescents between educational intervention groups and control groups [30]. Similar improvements have been observed across various populations, including adolescents [45], health care professionals [46], university students and academicians [47,48], and patients with osteoporosis [49]. Given the critical importance of maximizing peak bone mass during adolescence, implementing educational programs is highly justified [31].

Educational initiatives using diverse approaches and methods have consistently proven effective in increasing knowledge [45]. Web-based learning platforms offer flexible and accessible avenues for knowledge acquisition. Furthermore, leveraging technological advancements, such as those related to text, audio, and visuals, enhances information dissemination. This facilitates the development of interactive learning tools, such as images and videos, which effectively convey critical aspects of osteoporosis, such as nutrition and weight-bearing exercises [50].

### Practices Related to Bone Health

Despite significant gains in knowledge, this study did not observe notable improvements in dietary calcium intake and physical activity among adolescents after the intervention. Most adolescents (61/62, 98%) exhibited inadequate dietary calcium intake, with the median daily intakes before (median 576, IQR 473-688 mg/d) and after (median 586, IQR 491-755 mg/d) this study being below the recommended 1300 mg/d [16]. This trend aligns with findings from a previous study of Malaysian adolescents, in which all participants (N=794) had inadequate calcium intakes (calcium intake: mean 377.4, 95% CI 365.1-389.7 mg/d) [22]. Similar inadequate calcium intakes among adolescents have been reported globally, including in Morocco (calcium intake: mean 522, SD 297 mg/d) [51], Brazil (calcium intake: mean 618.2, 95% CI 570.8-665.5 mg/d) [52], and the United States (calcium intake: mean 962, SD 478 mg/d) [53], indicating a widespread issue of low dietary calcium intake among adolescents. Adolescents appear to consume limited amounts of calcium-rich foods, particularly dairy products [51].

This study also highlighted low physical activity levels among participants, with median scores of 2.2 (IQR 1.9-2.6) during the pretest and 2.3 (IQR 1.9-2.9) during the posttest. This finding is consistent with a previous study of 13-year-old adolescents (n=289) in Malaysia, where low physical activity levels were reported (mean 2.01, SD 0.03) [21]. Again, our findings align with global trends, showing that many adolescents do not meet recommended physical activity levels. For instance, the WHO reported that globally, 81% of adolescents aged 11 to 17 years are insufficiently physically active [54]. Similar trends were noted in Portugal [55], Sweden [56], China [57], and Korea [58], of which all indicated low physical activity levels among adolescents. For instance, Marques et al [55] reported that in a sample of 520,533 participants, less than 20% of adolescents in Portugal participated in physical activity every day, and nearly 20% never engaged in any form of physical activity. Sedentary behaviors and insufficient physical activity negatively impact bone health and overall well-being.

### Integrating Knowledge Into Behavior Changes

The gap between increased knowledge and unchanged health behaviors suggests that knowledge alone may not suffice to promote positive practices. Addressing both knowledge and bone health practices simultaneously is essential. This underscores the need for a comprehensive approach that integrates knowledge acquisition and behavior change techniques to foster optimal adolescent bone health practices. Our findings contrast with previous studies that demonstrated favorable impacts of educational interventions on behaviors such as calcium intake and physical activity among adolescents [59-61]. These disparities could be due to variations in the educational methodologies, particularly in facilitating behavior changes [59]. Direct educational approaches, such as interactive sessions that promote engagement and motivation, have shown promising results in improving adolescents’ nutritional practices and physical activity levels [49,60,62].

It is worth noting that our intervention lasted for only 2 weeks, whereas previous studies implemented interventions over 2 to 3 months [60-62]. Behavior changes often require an extended period to be effectively integrated into daily routines [63]. The transtheoretical model of behavioral change suggests that behavior modification is a dynamic and individualized process involving various stages, including precontemplation, contemplation, preparation, action, and maintenance [64]. Therefore, extending the intervention period is essential to fully observe and understand the intervention’s effects and assess its sustainability.

Nevertheless, a longer intervention alone does not guarantee sustainable behavior change. Incorporating behavior change theories and techniques, such as goal setting, self-monitoring, and feedback, may enhance the intervention’s effectiveness. These techniques could facilitate the translation of knowledge into sustained behavior changes by actively engaging adolescents in their lifestyle practices. Additionally, using interactive elements, such as videos, quizzes, and simulations within e-books, can enhance the learning experience and support effective behavior change [65]. To ensure the long-term sustainability of the intervention, future studies should include systematic follow-up assessments to measure the retention of bone health–related knowledge and behaviors. Identifying barriers and facilitators to the ongoing use of the e-book, such as user engagement and perceived relevance, is also essential.

### Acceptance Toward the e-Book

The e-book used in this study received positive feedback from adolescents, as they found it understandable, visually attractive, and well designed in terms of graphics and text size. Effective e-books should incorporate structured content and multimedia elements, such as sounds, graphics, images, text, and animations, to create an engaging and interactive learning experience [32]. e-Books can enhance adolescents’ understanding by using visual elements, including animated images, to convey the information more engagingly [66]. Incorporating interactive multimedia elements in e-books makes learning exciting, meaningful, and inspiring for adolescents [67]. Special animations and effects, such as graphics and sounds, effectively capture adolescents’ attention in educational settings.

### Strengths and Limitations

This study is the first to assess Malay adolescents’ knowledge and practices related to bone health following an educational intervention in Malaysia. The findings suggest that the e-book is effective for health promotion among adolescents. These results provide valuable insights for planning strategies to promote bone health among adolescents and the wider community, such as through the use of e-books.

Study limitations include the absence of a control group and uneven proportions of male and female respondents, which could have introduced bias. Additionally, the osteoporosis knowledge questionnaire and dietary calcium intake assessment evaluation tools have yet to be validated in the studied population. Furthermore, this study focused solely on calcium intake and physical activity, excluding other essential factors, such as vitamin D intake. The short intervention period also limited the ability to evaluate long-term behavior changes. Future studies should extend the intervention duration and include a broader range of bone health practices to provide a more comprehensive assessment.

### Recommendations for Future Studies

Future research should focus on enhancing the e-book’s content by incorporating more practical elements, such as easy-to-prepare recipes for calcium-rich foods and information on simple bone-health exercises. Integrating diversified and interactive educational content, including videos, quizzes, and simulations, can make learning more engaging and impactful. Such content can aim to engage adolescents and effectively promote the practical application of knowledge. To better foster sustained behavior changes, future studies should also consider extending the duration of the intervention period. Further, translating the e-book into other languages would increase its accessibility and cater to Malaysia’s diverse population. Moreover, conducting similar studies among non-Malay adolescents would offer a broader understanding of bone health practices across different ethnic groups in Malaysia.

### Conclusions

The developed e-book on bone health effectively increased knowledge related to bone health and was well received by Malay adolescents within our sample. However, the intervention did not significantly influence bone health practices, including dietary calcium intake (*P*=.31) and physical activity levels (*P*=.09). Additional strategies that integrate knowledge enhancement with practical demonstrations, interactive activities, and targeted approaches are necessary to promote the adoption of healthy behaviors for optimal bone health.

## Abbreviations

BMD: bone mineral density
FFQ: food frequency questionnaire
PAQ-A: Physical Activity Questionnaire for Adolescents
WHO: World Health Organization
